# Mediterranean dietary quality index and dietary phytochemical index among patients candidate for coronary artery bypass grafting (CABG) surgery

**DOI:** 10.1186/s12872-017-0544-z

**Published:** 2017-05-08

**Authors:** Mahdieh Abbasalizad Farhangi, Mahdi Najafi, Mohammad Asghari Jafarabadi, Leila Jahangiry

**Affiliations:** 1 Drug Applied Research Center, Nutrition Research Center, Faculty of Nutrition, Tabriz University of Medical Sciences, Tabriz, Iran; 2 Department of Research, Tehran Heart Center, Tehran University of Medical Sciences, North Karegar Street, Tehran, 1411713138 Iran; 3 Road Traffic Injury Research Center, Tabriz University of Medical Sciences, Tabriz, Iran; 4 Tabriz Health Services Managment Research Center, Tabriz University of Medical Sciences, Tabriz, Iran

**Keywords:** Med-DQI, Mediterranean dietary style, Dietary phytochemical index, CABG

## Abstract

**Background:**

The aim of the present research was to evaluate the relationship between Mediterranean dietary quality index (Med-DQI) and dietary phytochemical index (DPI) with metabolic risk factors of cardiovascular disease in candidates for coronary artery bypass graft (CABG) surgery.

**Methods:**

This was a cross-sectional study on 454 patients aged 35–80 years as candidates of CABG and hospitalized in Tehran Heart Center. Anthropometric and demographic characteristics were obtained from all participants and a 138-item semi-quantitative food frequency questionnaire (FFQ) was used to evaluate Med-DQI and DPI. Biochemical parameters including HbA1C, serum lipids, albumin, creatinine and C-reactive protein (CRP) were assessed by commercial laboratory methods.

**Results:**

Patients with higher scores of “saturated fatty acids” had lower serum albumin concentrations (*P* < 0.05). High scores of “cholesterol” subgroup was also accompanied with higher serum Hb A1C percent (*P* = 0.04). Significantly higher concentrations of serum creatinine were also observed in categorizes with lower “fish” scores. Patients with lower phytochemical intakes had significantly higher Med-DQI scores.

**Conclusion:**

According to our findings, high dietary intakes of saturated fatty acids and cholesterol were associated with low serum albumin and Hb A1C concentration. Further studies are needed to better clarify these associations and possible underlying mechanisms.

## Backgrounds

Cardiovascular disease (CVD) is one of the most common causes of morbidity and mortality in different communities accounting for more than 31% or 17.5 million deaths worldwide; more that 75% of these deaths occur in low and middle income countries and nearly 50% of all deaths in Iran [1, 2]. Coronary artery bypass grafting (CABG) is the most common type of open-heart surgical interventions for the treatment of patients in the higher stages of coronary artery disease (CAD), where atherosclerosis of one or more of coronary arteries is severe enough to show at least 50% stenosis of arterial lumen in angiographic image. The number of CABG operations carried out to treat CAD has increased more than fivefold since 1980, and the general trend has been an almost steady rise in the number of operations performed each year [3]. Independent risk factors of CVD include a family history of premature coronary artery disease, cigarette smoking, diabetes mellitus, hypertension, dyslipidemia, sedentary life style, unhealthy food choices and poor eating habits [4].

The role of dietary factors and nutritional regimens in prevention of CVD and its progression has been extensively studied; numerous reports suggested the role of healthy dietary choices and improved life style with higher physical activity level [5] and higher intakes of healthy food choices including fruits and vegetables and dietary antioxidants in prevention and treatment of cardiovascular events [6].

The Mediterranean diet is considered as one of the healthiest dietary models, and numerous epidemiological and nutritional studies have shown that Mediterranean countries benefit from lower rates of morbidity from chronic disease and higher life expectancy [7]. The Mediterranean dietary pattern is characterized by a high intake of vegetables, legumes, fruits and nuts, cereals (that in the past were largely unrefined), a high intake of olive oil but a low intake of saturated lipids, a moderately high intake of fish, a low-to-moderate intake of dairy products (and then mostly in the form of cheese or yoghurt), a low intake of meat and poultry and a regular but moderate intake of alcohol [8]. The diet exerts most of its health-promoting effects via its bioactive compounds including phytochemicals. These phenolic ingredients mostly concentrated in olives, grapes and nuts protect against cardiovascular events, oxidative stress and vascular dysfunction [9, 10]. The Mediterranean diet is the best example of a phytochemical-rich diet; recently, two studies have reported that Mediterranean and phytochemicals-rich diets both reduce total cholesterol, LDL-C and non-HDL-C levels and has significant cardio-protective effects [11]. Dietary phytochemical content can be evaluated by dietary phytochemical index (DPI) first proposed by McCarty [12]. DPI, defined as the percent of dietary calories derived from foods rich in phytochemicals could be used as an index of total dietary phytochemical content; this index is a simple method for assessment of phytochemical intake that, despite its limitations, could provide important background for diet quality and may have high practical and clinical uses [12, 13].

Several previous studies suggested the protective role of Mediterranean dietary regimen in prevention of cardiovascular events [6] and other chronic disease like diabetes and metabolic syndrome [6, 14]. In a multi- center trial in Spain, a Mediterranean diet supplemented with extra-virgin olive oil and a Mediterranean diet supplemented with mixed nuts reduced the risk of major cardiovascular disease by 30% and 28% respectively [6]. In other study by Hoscan et al. in Turkish population, for each score of reduction in Mediterranean diet intake, in men, the risk of myocardial infarction, coronary bypass, coronary angioplasty, and any cardiovascular disease in men increased by 1.3 (*P* = 0.02), 1.4 (*P* = 0.03), 1.5 (*P* = 0.01), and 1.3 (*P* = 0.02) respectively, while in women, the risk of myocardial infarction and angioplasty increased by 1.3 (*P* = 0.02) and 1.5 (*P* = 0.01), respectively [15]. In a meta-analysis by Martinez-Gonzalez MA [16], an intervention with a Mediterranean diet was associated with a 38% relative reduction in the risk of CVD clinical events (pooled random-effects risk ratio: 0.62; 95% confidence interval, CI: 0.45–0.85). It has been suggested that there would be a synergy among the nutrient-rich foods included in the Mediterranean diet that fosters favorable changes in intermediate pathways of cardio-metabolic risk factors, like blood lipids, insulin sensitivity, resistance to oxidation, inflammation, and vaso-reactivity [17].

Almost all of the above-mentioned studies focused on evaluating the relations between Mediterranean dietary pattern and the risk of disease. However, findings based on dietary patterns that depend on the consumption characteristics of the sample under study cannot be generalized; In fact, based on real cultural heritage and traditions, a priori indices like Mediterranean dietary scores, used to evaluate adherence to the Mediterranean diet should consider classifying whole grains and refined grains olive oil and monounsaturated fats, and wine and alcohol differently [18]; Mediterranean dietary quality index (Med-DQI) first developed by Gerber et al. [19] is a useful tool to evaluate quality of diet highlighting two different sources of fat (saturated and olive oil) and two different sources of protein (meat and fish) with the opposite scores, one on the poor side and other on the good side respectively (Table 1). There are some studies assessing dietary quality in patients with myocardial infarction [20] or established CAD [21] in which a priori healthy diet pattern score was used. However, to our review of literature, no report was found evaluating the Mediterranean dietary quality index or dietary phytochemical index in patients undergoing CABG surgery in Iran. Therefore in the current study we aimed to investigate Med-DQI and DPI in CABG patients during 1 year pre-operation period, and looked for associations with some demographic factors and biochemical risk factors.

**Table 1:** Construction of the score for the Mediterranean Dietary Quality Index

Scoring	0	1	2
Saturated fatty acids (% energy)	<10	10–13	>13
Cholesterol (milligram)	<300	300–400	>400
Meats (gram)	<25	25–125	>125
Olive oil (milliliter)	>15	15–5	<5
Fish (gram)	>60	60–30	<30
Cereals (gram)	>300	300–100	<100
Vegetables + fruits (gram)	>700	700–400	<400

## Methods

### Subjects

Participants in the current cross-sectional study were candidates for isolated CABG with cardiopulmonary bypass and were recruited for Tehran Heart Center-Coronary Outcome Measurement (THC-COM) study. The study was carried out between May–September 2006. Participants in this study were patients admitted to the cardiothoracic ward for CABG surgery at a large Heart Center in this time period (Tehran heart center, Iran). The sample size calculation has been explained before [22]; briefly, the sample size was calculated using the formula for comparing two proportions: n = [(Zα/2 + Zβ)2 × {(p_1_ (1-p_1_) + (p2 (1-p_2_))}]/ (p_1_ - p_2_)^2^ where p_1_ is the proportion of the women with low quality Mediterranean regimen (0.3), p_2_ is the proportion of the men with low quality Mediterranean regimen (0.25), α error = 0.05, and power = 80% (1-β). Accordingly, a 125-subject sample size was determined for the study (125 in each group). We also assumed 20% loss (125 + 25) and as men with CAD are twice as women (150 + 300), the final sample size of 450 was considered for the study [22, 23]. Reasons for drop-out or exclusion were incomplete dietary questionnaires (*n* = 1), and incomplete demographic questionnaires (*n* = 5). The final analytic sample in this study consisted of 454 patients aged 35–80 years who completed both the questionnaire and the medical examination. More details of study procedure and biochemical assays have been provided elsewhere [22]. Written informed consent was obtained from each participating subject. The study was approved by Tehran Heart Center, Tehran University of Medical Sciences.

### Dietary assessment methods

A 138-item semi-quantitative food frequency questionnaire (FFQ) was used to assess the habitual dietary intakes of patients. The FFQ consisted of a list of foods with standard serving sizes commonly consumed by Iranians. Participants were asked to report how often they consumed each of the food items listed as the number of times per day, per week, per month or per year during the previous year. The reported frequency for each food item was then converted to a daily intake. Portion sizes of consumed foods were converted to grams by using household measures [24]. The questionnaire was previously validated for healthy Iranian population [25].

We calculated the diet score on the basis of Mediterranean diet quality index (Med-DQI) (Table 1). The index assigns a score of 0, 1 or 2 according to the daily intake of each of the seven components and then final score was reported as a summation of all nutrient scores ranged between 0 and 14. A lower score on this index denotes a better nutrition quality and higher adherence to Mediterranean dietary pattern [19].

The dietary phytochemical index (DPI) was defined as the percent of dietary calories derived from foods rich in phytochemicals. Calories derived from fruits, vegetables (except for potatoes), legumes, whole grains, nuts, seeds, fruit/vegetable juices, soy products, wine, beer, and cider are enumerated in this index. The higher score denotes the higher phytochemical content of diet [12].

### Statistical analysis

Data analysis was performed by SPSS statistical software package version 16 (SPSS Inc., Chicago, IL, USA). Kolmogorov–Smirnov test was performed for normality of the distributions of variables. The comparison of discrete variables was performed by Chi- square test. Continuous variables between groups were compared by independents sample- *t* test or one way analysis of variance (ANOVA). Analysis of covariance (ANCOVA) was used to compare continuous variables between three groups adjusting for the confounding effects of age, gender and body mass index. General linear model was also applied for evaluating the association between total Med-DQI score and biochemical parameters adjusting for the mediating effects of dietary phytochemical score. *P* values less than 0.05 considered as significance level.

## Results

As mentioned previously, higher MED-DQI scores denote higher adherence to Mediterranean dietary pattern and better nutrition quality. While higher DPI score indicates higher intake of dietary phytochemicals. Patients general information according to different categorizes of Med-DQI and DPI are presented in Table 2. Mean age and BMI were not significantly different between two categorizes of Med-DQI. However patients with lower Med-DQI score were more likely to be male and have higher educational attainment (*P* < 0.05). Additionally the prevalence of diabetes was lower in the higher scores of Med-DQI. Patients in higher tertiles of dietary phytochemical index had higher BMI and were most likely to be male (*P* < 0.05).

**Table 2:** General characteristics of study participants according to different categorizes of total Med-DQI and DPI scores

	Med-DQI score		*P* ^†^ value	DPI tertiles			*P* value^‡^
	<6	> 6		1st	2nd	3rd	
Age (y)	58.99 ± 8.93	58.27 ± 3.80	0.95	58.45 ± 9.92	59.52 ± 8.61	59.04 ± 8.38	0.59
Male (%)	233 (77.2)	100 (65.8)	*0.004*	125 (83.9)	100 (67.6)	108 (68.8)	*0.016*
Body mass index (kg/m^2^)							
*<24.9*	82 (27.7)	48 (32.2)	0.28	52 (35.6)	31 (21.2)	37 (30.7)	*0.02*
*25–29.9*	132 (44.6)	65 (43.6)		69 (47.3)	71 (48.6)	57 (37.3)	
*≥ 30*	82 (27.7)	36 (24.2)		25 (17.1)	44 (30.1)	49 (32.0)	
Educational level (%)							
*Uneducated*	148 (50.5)	93 (62.4)	*0.02*	77 (52.7)	88 (60.7)	76 (50.3)	0.34
*< Diploma*	96 (32.8)	39 (26.2)		51 (34.9)	36 (24.8)	48 (31.8)	
*Diploma and higher*	49 (16.7)	17 (11.4)		18 (12.3)	21 (14.5)	27 (17.9)	
Diabetes (%)	119 (39.4)	73 (48.0)	*0.04*	60 (40.3)	62 (41.9)	70 (44.6)	0.44
Hyperlipidemia (%)	219 (72.5)	104 (68.4)	0.21	104 (69.8)	109 (73.6)	110 (70.1)	0.69
Hypertension (%)	145 (48.2)	72 (47.4)	0.47	65 (43.6)	75 (50.7)	77 (49.4)	0.32

^†^ *P* values from independent sample T-test, ^‡^ *P* values from ANOVA analysis. The comparison of discrete variables is performed by χ^2^ test. The significant *P* values are presented as italic

**Fig. 1:**
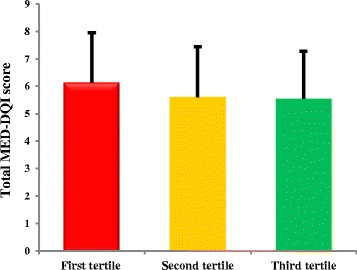
Mediterranean dietary quality index among different tertiles of dietary phytochemical scores (*P* values of ANOVA test < 0.001)

Table 3 presents the comparison of laboratory parameters according to components of Mediterranean dietary quality index in patients. As shown in this Table, patients with higher intakes of “saturated fatty acids” had lower serum albumin concentrations (*P* < 0.05). High scores of “cholesterol” subgroup was also accompanied with higher serum Hb A1C percent (*P* = 0.04) denoting the higher likelihood of diabetes by high intake of dietary cholesterol. Significantly higher concentration of serum creatinine was also observed in categorizes with higher “fish” intakes; while serum lipoprotein (Lp) (a) concentration was increased in top scores of “cereals”. In other word, lower cereal intake was associated with higher serum Lp (a) concentrations (*P* = 0.05). In General linear model for evaluation of the association between total Med-DQI score and biochemical parameters adjusting for the mediating effects of DPI, total Med-DQI score was associated with serum CRP and creatinine concentrations and these associations remained significant even after adjusting for the mediating effects of DPI (Table 4). Moreover, patients with lower dietary phytochemical intake had significantly higher Med-DQI scores (Fig. 1, *P* < 0.004).

**Table 3:** Comparison of laboratory parameters according to components of Mediterranean dietary quality index in patients candidate for CABG

Characteristics									
	Hb A_1_C (%)	TG(mg/dl)	TC(mg/dl)	HDL(mg/dl)	LDL(mg/dl)	CRP(mg/dl)	Albumin (g/dl)	Creatinine (mg/dl)	Lipoprotein (a) (mg/dl)
Saturated fatty acid (n)									
0 (216)	6.05 ± 1.72	172.48 ± 82.66	158.27 ± 43.66	40.19 ± 8.61	85.52 ± 40.88	6.84 ± 0.87	4.69 ± 0.36	1.33 ± 0.29	31.43 ± 24.76
1 (163)	6.20 ± 1.85	176.46 ± 99.20	162.85 ± 48.75	40.90 ± 9.14	88.05 ± 39.15	6.58 ± 0.25	4.63 ± 0.32	1.27 ± 0.27	34.18 ± 27.15
2 (63)	6.07 ± 1.57	179.28 ± 92.85	165.09 ± 44.94	40.53 ± 9.20	89.35 ± 38.88	6.89 ± 0.69	4.59 ± 0.25	1.26 ± 0.20	31.88 ± 29.37
P value*	0.73	0.83	0.45	0.74	0.72	0.87	*0.04*	0.08	0.58
Cholesterol (n)									
0 (318)	6.01 ± 1.65	173.03 ± 1.12	160.89 ± 43.89	40.42 ± 8.56	87.95 ± 40.74	6.64 ± 5.22	4.65 ± 0.32	1.28 ± 0.28	33.32 ± 26.85
1 (66)	6.14 ± 1.74	178.76 ± 80.16	163.33 ± 49.12	40.81 ± 10.22	87.74 ± 41.45	6.63 ± 3.40	4.62 ± 0.34	1.39 ± 0.29	30.21 ± 23.38
2 (58)	6.63 ± 2.15	181.18 ± 97.78	158.46 ± 52.26	40.61 ± 9.16	80.80 ± 32.82	7.51 ± 7.25	4.74 ± 0.35	1.28 ± 0.20	30.40 ± 26.67
P value*	*0.04*	0.76	0.83	0.94	0.45	0.51	0.11	0.68	0.57
Meats (n)									
0 (16)	6.03 ± 1.56	164.09 ± 80.62	171.49 ± 51.21	39.98 ± 7.93	89.53 ± 23.43	5.65 ± 2.06	4.65 ± 0.30	1.28 ± 0.21	22.28 ± 12.29
1 (313)	6.03 ± 1.62	171.88 ± 80.49	161.93 ± 45.14	40.84 ± 8.73	87.92 ± 39.62	6.55 ± 5.15	4.65 ± 0.34	1.29 ± 0.27	32.31 ± 25.63
2 (113)	6.33 ± 2.07	184.96 ± 114.19	156.58 ± 46.51	39.65 ± 9.43	84.10 ± 42.75	7.46 ± 5.93	4.67 ± 0.31	1.33 ± 0.28	34.63 ± 29.38
P value*	0.27	0.35	0.34	0.45	0.65	0.20	0.78	0.32	0.17
Olive oil (n)									
0 (52)	6.09 ± 1.53	164.07 ± 79.41	155.09 ± 40.85	41.88 ± 8.82	87.86 ± 58.85	5.57 ± 1.30	4.70 ± 0.36	1.30 ± 0.24	30.85 ± 24.81
1 (136)	6.03 ± 1.53	184.01 ± 104.87	160.25 ± 40.84	40.32 ± 8.79	84.19 ± 34.46	6.50 ± 3.49	4.66 ± 0.29	1.33 ± 0.31	32.82 ± 25.31
2 (254)	6.15 ± 1.89	172.33 ± 83.81	162.49 ± 49.07	40.32 ± 8.95	88.31 ± 37.93	7.13 ± 6.45	4.65 ± 0.34	1.28 ± 0.25	32.66 ± 27.23
P value*	0.82	0.31	0.55	0.48	0.61	0.12	0.53	0.15	0.88
Fish (n)									
0 (57)	6.20 ± 2.32	163.62 ± 80.35	158.86 ± 45.51	40.40 ± 8.22	91.34 ± 57.50	5.30 ± 0.69	4.64 ± 0.32	1.39 ± 0.34	32.49 ± 27.09
1 (91)	6.09 ± 1.56	188.57 ± 107.92	156.73 ± 36.06	39.72 ± 9.10	82.02 ± 27.85	4.29 ± 0.44	4.70 ± 0.32	1.30 ± 0.25	33.74 ± 29.66
2 (294)	6.09 ± 1.68	172.94 ± 86.01	162.62 ± 48.38	40.76 ± 8.95	87.69 ± 38.92	5.58 ± 0.34	4.64 ± 0.34	1.28 ± 0.26	32.12 ± 25.14
P value*	0.91	0.21	0.52	0.61	0.33	0.62	0.31	*0.02*	0.41
Cereal (n)									
0 (315)	6.11 ± 1.80	179.55 ± 95.83	159.84 ± 44.89	40.18 ± 8.73	6.39 ± 3.49	4.67 ± 0.32	1.31 ± 0.26	31.78 ± 24.57	39.61 ± 11.44
1 (119)	6.12 ± 1.62	162.60 ± 73.23	161.77 ± 46.83	41.17 ± 9.27	7.78 ± 8.42	4.64 ± 0.36	1.27 ± 0.31	35.51 ± 30.71	42.17 ± 13.74
2 (8)	5.66 ± 1.23	174.88 ± 88.19	192.62 ± 57.75	43.50 ± 8.83	5.73 ± 0.69	4.47 ± 0.26	1.32 ± 0.08	16.12 ± 14.61	45.62 ± 12.78
P value *	0.76	0.21	0.13	0.36	0.11	0.44	0.23	0.45	*0.059*
Fruits and vegetables (n)									
0 (369)	6.10 ± 1.73	175.64 ± 94.06	160.17 ± 43.46	40.62 ± 9.31	39.69 ± 2.04	6.65 ± 5.28	4.66 ± 0.33	1.31 ± 0.28	31.29 ± 25.22
1 (62)	6.16 ± 1.93	166.79 ± 64.37	162.13 ± 56.29	40.37 ± 6.48	40.79 ± 5.09	7.44 ± 5.84	4.62 ± 0.33	1.25 ± 0.22	39.89 ± 31.82
2 (11)	5.94 ± 1.35	197.82 ± 90.95	180.27 ± 54.44	37.18 ± 4.16	42.11 ± 12.69	6.55 ± 1.64	4.71 ± 0.17	1.14 ± 0.17	32.55 ± 25.00
P value*	0.92	0.53	0.34	0.44	0.33	0.55	0.58	0.09	0.06

*P values from ANCOVA adjusted for the confounding effects of age, gender and body mass index. The significant *P* values are presented as italic

**Table 4:** General Linear Model (GLM) for evaluating the association between total MED-DQI score and biochemical parameters with and without mediating effects of DPI

	With DPI as co-variate		Without DPI as co-variate	
	F	P value*	F	P value*
Hb A_1_C (%)	0.84	0.58	0.85	0.58
TG(mg/dl)	0.51	0.88	0.51	0.88
TC(mg/dl)	1.2	0.28	1.2	0.24
HDL (mg/dl)	1	0.44	0.99	0.44
LDL(mg/dl)	1.25	0.25	1.23	0.27
CRP(mg/dl)	1.72	*0.05*	1.71	*0.049*
Albumin(g/dl)	1.58	0.11	1.65	0.089
Creatinine (mg/dl)	2.46	*0.007*	2.29	*0.012*
Lipoprotein (a) (mg/dl)	1.37	0.19	1.36	0.19

*P*-values obtained from general linear model with and without mediating effects of DPI. The significant *P* values are presented as italic

## Discussion

Adherence to Mediterranean diet lowers the risk of coronary artery events and reduces the risk myocardial infarction, coronary bypass graft, percutaneous coronary intervention and coronary artery disease rates [15]; on the other hand, patients candidate for CABG, did not have an acceptable nutritional status before and after surgery and malnutrition is a common feature of CABG [26, 27]. Therefore, having a good nutritional status before surgery can be a useful strategy for prevention of CABG-induced malnutrition. Therefore it is very important to develop healthy dietary regiments for improving the patients’ nutritional status and quality of life.

Numerous evidences suggested the protective role of Mediterranean dietary pattern against cardiovascular disease [28] and its associated risk factors including metabolic syndrome, diabetes and obesity [29, 30]. In fact, over the past decades, numerous dietary models have been proposed to protect against metabolic abnormalities and among them, only the Mediterranean diet demonstrated a beneficial effect [31].

In the current study we demonstrated that patients with lower Med-DQI scores had higher educational attainment compared with patients with higher scores. Moreover, patients with higher scores of “saturated fatty acids” and “cholesterol” had significantly lower serum albumin and creatinine concentrations and higher Hb A1C percent respectively.

In a sub-analysis of the EPIC study analyzed a cohort of 497,308 people showed that a higher adherence to the Mediterranean diet was associated with a significantly lower body mass index and waist circumference within 3 years [29]. Likewise, the beneficial role of Mediterranean dietary pattern in protecting against CVD risk factors has been supported by several studies. A two point increase of Mediterranean dietary score was associated with 33% reduced risk of mortality from cardiovascular causes (RR = 0.67, 95%, CI: 0.47–0.94) [28]. The role of Mediterranean dietary style in primary prevention of CVD in a sample of 7747 adults at high risk of CVD but without a manifest disease has been established in a large interventional study before [6].

The Mediterranean dietary quality index (Med-DQI) is a useful tool for predicting dietary quality and has been validated previously using nutritional biomarkers [19]. This index was based on the recommendations made by the National Research Council and American Heart Association regarding the diet and health [32]. These recommendations are based on consumption of 30% or less of the daily total energy from fat, 10% or less of the total energy from saturated fat, 30 mg/d or less from cholesterol, 55% of energy from complex carbohydrates and 5 servings or more from fruits and vegetables.

In the current study, for the first time, we evaluated the Med-DQI in patients undergoing CABG and according to our findings, higher scores of “saturated fatty acid” was associated with lower serum albumin concentrations (*P* = 0.04). Albumin is a main fatty acid transporter in the extracellular fluids and dietary factors influence its plasma concentrations [33]. Several animal studies observed the albumin- lowering effects of high fat diets. Andrson et al. [34] found that feeding high fat diet in mice induces a slight decrease in serum albumin concentrations. Since Albumin synthesis is sensitive to insulin, they speculated that reduced serum albumin concentrations and biosynthesis is a result of high fat diet- induced insulin resistance. In other study, female rats feeding high-fat diets had 25% lower serum albumin concentrations [35].

Interestingly, patients in the lower categorizes of “cholesterol” subgroup had significantly higher serum HbA_1_C concentrations. Hb A1C is a better predictor of circulating lipids and better diagnostic marker of developing cardiovascular and micro-vascular complications [36, 37]. Previous studies observed a positive association between dietary cholesterol intake and risk of type 2 diabetes mellitus [38, 39]. Since cholesterol is only present in products of animal origin, one can speculate that these associations could represent an adverse effect of a food pattern characterized by a high consumption of meat and eggs, or even an unidentified component of animal products [40]. However dietary cholesterol could have direct effects in incidence of type 2 diabetes via stimulating inflammatory pathways [41]. In animal models, dietary cholesterol per se produces an increase in serum amiloid A, a potent inflammatory mediator [42]. These inflammatory mediators can lead to incidence of type 2 diabetes mellitus [43]. Moreover, dietary cholesterol disrupts glucose metabolism and increases serum insulin concentrations [38].

Serum creatinine is a marker of muscle mass and also dietary protein intake [44]. High dietary protein can also significantly raise serum creatinine concentrations [45]. In our study, patients with higher fish intake had higher serum creatinine concentrations, although none of these differences were out of normal range and these variations occurred in the physiologically normal range of serum creatinine. Previous studies also confirm our findings. In the study by Rasmussen et al. the higher dietary fish intake was associated with higher urinary creatinine excretion [46].

We also observed high serum Lp (a) concentrations in patient with low intakes of cereals. Accordingly, the beneficial effects of cereals on serum Lp (a) have been previously confirmed. In the study by Xiong et al. breakfast and bran cereals had potent positive effects on reduction of serum Lp (a) concentrations [47]. Lp (a) is essentially an LDL particle with an apoprotein (a) attached to it. Numerous evidences have revealed that high serum Lp (a) concentration is associated with a variety of CVD events, including peripheral vascular disease, cerebrovascular disease, premature coronary disease and vascular injury [48]. The impact of cereals in improving serum lipoproteins and lipids are attributed to their fiber content as previously confirmed [49].

## Conclusions

The present study reported that patients with higher scores of “saturated fatty acids” and “cholesterol” had significantly lower serum albumin and higher Hb A1C percent respectively. Higher intake of “fish” was also associated with higher serum creatinine concentration. Further studies with interventional approaches are needed to better clarify the causal inference of these associations.

## Abbreviations

ANCOVA: Analysis of co-variance
BMI: Body mass index
CABG: Coronary artery bypass grafting
CRP: C-reactive protein
CVD: Cardiovascular disease
FBS: Fasting blood sugar
FFQ: Food frequency questionnaire
HC: Hip circumference
HDL-C: High density lipoprotein cholesterol
LDL-C: Low density lipoprotein cholesterol
MED-DQI: Mediterranean dietary quality index
TC: Total cholesterol
TG: Triglyceride
THC-COM: Tehran heart center-coronary outcome measurement
WC: Waist circumference
WHR: Waist to hip ratio
